# The dataset of de novo transcriptome assembly of *Falcataria moluccana* cambium from gall-rust (*Uromycladium falcatarium*) infected and non-infected tree

**DOI:** 10.1016/j.dib.2019.104489

**Published:** 2019-09-07

**Authors:** Hasyyati Shabrina, Ulfah J. Siregar, Deden D. Matra, Iskandar Z. Siregar

**Affiliations:** a Tropical Silviculture Program, Graduate School, IPB University (Bogor Agricultural University), Indonesia; b Department of Silviculture, Faculty of Forestry, IPB University (Bogor Agricultural University), Indonesia; c Department of Agronomy and Horticulture, Faculty of Agriculture, IPB University (Bogor Agricultural University), Indonesia

**Keywords:** *De novo*, *Falcataria moluccana*, Gall-rust (*Uromycladium falcatarium*) infected, Next-generation sequencing (NGS), RNA, Transcriptome

## Abstract

Sengon (*Falcataria moluccana*), formerly known as *Albizia falcataria* or *Paraserianthes falcataria*, is an essential tree species for the development of community-based timber plantation, especially in Indonesia. The plantations nowadays are facing a significant disease threat, namely infection of gall-rust fungi (*Uromycladium falcatarium*). However, a clear understanding of the molecular mechanisms of the tree response against the disease is still unknown. We carried out transcriptome assembly using BGISEQ-500 technology to provide assembled de novo transcriptome dataset generated from gall-rust infected and non-infected trees. The construction of assembled transcriptome was conducted using Trinity v.2.3.2 The raw reads are available in the DDBJ platform with accession number, DRA007983.

**Table:** Specifications Table

Subject	Agricultural and Biological Sciences: Forestry
Specific subject area	Molecular study in Forestry
Type of data	RNA Sequencing Data
How data were acquired	The data acquired by Next-generation Sequencing technology using BGISEQ-500 platform
Data format	Raw Sequencing reads and assembled contigs
Parameters for data collection	The cambium was collected each from outer sapwood of gall-rust infected and non-infected Sengon tree.
Description of data collection	The RNA was sequenced with Next Generation Sequencing method using BGISEQ-500 platform in Beijing Genomic Institute, Hongkong.
Data source location	City/Region: Bogor/West Java Country: Indonesia Latitude and longitude (and GPS coordinates) for collected samples/data: 6°32′38.9″S 106°44′24.5″E
Data accessibility	Repository name: DDBJ (DNA Data Bank of Japan) Data identification number: DRA007983 Direct URL to data: https://ddbj.nig.ac.jp/DRASearch/submission?acc=DRA007983
Related research article	N.E. Lelana, S. Wiyanto, Gianto, I.Z. Siregar. Genetic diversity of *Falcataria moluccana* and its relationship to the resistance of gall rust disease. Biodiversitas (2018): 12–17. DOI: https://doi.org/10.13057/biodiv/d190102 D. Rahmawati, N. Khumaida, U.J. Siregar. Morphological and phytochemical characterization of susceptible and resistant Sengon (*Falcataria moluccana*) tree to gall rust disease. Biodiversitas 20 (2019): 907–913. DOI: https://doi.org/10.13057/biodiv/d200340

**Table:** 

**Value of the data** • This data provides the assembled-transcriptome of Sengon (*Falcataria moluccana*) from cambium tissue • This data will be of practical use to develop genetic markers as a tool for assisting gall-rust resistant Sengon tree improvement program • This data will further be advantageous in analyzing differential gene expression to understand molecular mechanism regarding infection of gall rust disease in Sengon

## 1 Data

This data presented the de novo transcriptome assembly of *Falcataria moluccana* (Miq.) Barneby & JW Grimes (Indonesia: Sengon, Malaysia: Batai, Hawaii: Albizia, East Timor: Madre de Cacao, Philippines: Falcata). The Sengon tree is cultivated widely in Indonesia as a material source for veneer, light construction, and pellet in bioenergy. However, the plantations are facing the most damaging threat that damaging the wood and lowering the productivity, gall-rust (*Uromycladium falcatarium*) fungi infection. Altough the fungi could infect any tissue, the wood or stem, which is the main product of the plantation, was the part that damaged badly. After the fungi entered the bark, the gall-rust fungi will wounded the cell in cambium, causing death in some cells and grow itself in cambium tissues by blocking the nutrient. The fungi also secretes growth hormones that caused the undifferentiated cell in cambium to grow rapidly and created wood deformity. The transcriptome of Sengon was derived from the RNA collected from the cambium part of the stem. The sequencing process was generated using BGISEQ-500 platform. The properties of the reads and the assembled sequences are shown in Table 1. Overview of the transcriptome data is presented in Table 2. The KEGG pathway analysis generated pathways with high number of contigs which involved in gall-rust infection incidence (Table 3). Among the microsatellite motifs from the merged sequences (Table 4), mono- (27,917; 39.83%) and dinucleotide (8,242; 11.76%) were the most common, and the three most frequent motifs were A/T (26,157; 64.33%), AG/CT (4,300; 10.58%), and AT/AT (2,195; 5.40%). The most frequent trinucleotide, tetranucleotide, pentanucleotide, and hexanucleotide were AAG/CTT (1,378; 3.39%), AAAG/CTTT (83; 0.2%), AAAAG/CTTTT (8; 0.02%), and AAACCC/GGGTTT (7; 0.02%) respectively.

**Table 1:** The properties of raw reads and assembled sequences of Sengon cambium.

Features	Numbers		
	infected	non-infected	Merged
Raw Reads			
Numbers	79,054,112	79,037,484	158,090,000
Bases	7,905,411,200	7,903,748,400	15,810,000,000
Unique Transcripts			
Numbers	380,032	118,171	400,633
Bases	278,860,152	114,550,451	325,278,365
Length range (bp)	201-17,824	201-8,098	201-12,634
Average (bp)	733.78	969.36	811.91
N50 (bp)	1324	1439	1521
GC contents (%)	40.1	40.6	39.8
Contigs			
Numbers	72,968	41,332	70,089
Bases	100,689,847	57,231,623	115,068,922
Length range (bp)	201-21,986	201-8,098	181-15,997
Average (bp)	733.78	1385	1641
N50 (bp)	1074	1243	1382
GC contents (%)	41.1	40.8	40.6

**Table 2:** Functional annotation of Sengon contigs.

Source	Number (percentage)		
	Infected	Non-infected	Merged
Contig Number	72,968	41,332	70,089
Contigs:			
Nr NCBI	–	–	68,927 (98.34)
Nt NCBI	61,213 (83.89)	37,986 (91.90)	62,880 (89.71)
SwissProt	40,694 (55.76)	25,633 (62.02)	42,783 (61.04)
TrEMBL	–	–	58,593 (83.59)
Gene ontology	–	–	17,134 (24.45)
KEGG	–	–	3,256 (4.65)

**Table 3:** Top 20 KEGG pathway with the highest contig numbers of merged sequence.

Pathway	Pathway ID	#Enzymes in pathway	#Contigs of Enzyme
Purine metabolism	map00230	7	54
Thiamine metabolism	map00730	2	44
Biosynthesis of antibiotics	map01130	8	31
Pyrimidine metabolism	map00240	8	17
Folate biosynthesis	map00790	2	16
One carbon pool by folate	map00670	3	16
beta-Lactam resistance	map01501	1	13
Drug metabolism - other enzymes	map00983	2	13
Penicillin and cephalosporin biosynthesis	map00311	1	13
Terpenoid backbone biosynthesis	map00900	1	12
Aminoacyl-tRNA biosynthesis	map00970	3	9
Alanine, aspartate and glutamate metabolism	map00250	3	8
Cysteine and methionine metabolism	map00270	3	8
Porphyrin and chlorophyll metabolism	map00860	3	6
Galactose metabolism	map00052	1	6
Oxidative phosphorylation	map00190	1	5
Glutathione metabolism	map00480	2	4
Pantothenate and CoA biosynthesis	map00770	3	4
Cyanoamino acid metabolism	map00460	1	3
Glycine, serine and threonine metabolism	map00260	2	3
Phenylalanine, tyrosine and tryptophan biosynthesis	map00400	2	3

**Table 4:** The number and motif of microsatellite of Sengon contigs.

Motifs	Number of contigs (percentage)		
	Infected	Non-infected	Merged
Mononucleotide	15,979 (21.90)	18,786 (45.45)	27,917 (39.83)
Dinucleotide	7,157 (9,81)	3,879 (9.38)	8,242 (11.76)
Trinucleotide	3,676 (5.04)	2,258 (5.46)	4,172 (5.95)
Tetranucleotide	216 (0.30)	94 (0.23)	255 (0.36)
Pentanucleotide	31 (0.04)	6 (0.01)	28 (0.04)
Hexanucleotide	41 (0.06)	22 (0.05)	48 (0.07)

## 2 Experimental design, materials, and methods

The cambiums of Sengon tree were collected from a private plantation in Bogor, West Java, Indonesia (6°32′38.9″S 106°44′24.5″E). The RNA was extracted from the cambium part originated from gall-rust infected and gall-rust free tree, each type of tree consist of one individual and no replication. The RNA extracted by BGI from 80 mg tissue sample using established CTAB-pBIOZOL method. The integrity and quantity of isolated-RNA were monitored and quantified by a NanoDrop ND-1000 spectrophotometer and Agilent 2100 Bioanalyzer. The non-infected sample was treated with Ribozero due to ribosomal RNA contamination. The RNA sequencing was carried out using the BGISEQ-500 (BGI, Hong Kong) and produced pre-processing reads to discard the adaptors and low-quality reads. The pre-processed reads were de novo assembled by Trinity v.2.3.2 [1], and the high-quality contigs were obtained [2], [3]. These contigs were annotated using BLAST + program [4] against the NCBI non-redundant (Nr), Nucleotide sequences (Nt) and protein sequence database of UniProt (SwissProt and TrEMBL). The annotated-contigs from Nr database were performed using Blast2Go software [5] to obtain functional annotation of Gene Ontology and KEGG pathway. Identification of contigs containing microsatellites was performed using MISA program [6], with minimum repeats were: 10 for 1 base, 6 for 2 bases, and 5 for 3, 4, 5, and 6 bases; and the interruptions (the maximum difference between microsatellites) were 100 bases.

## References

[1] Grabherr M.G. (2011). Full-length transcriptome assembly from RNA-Seq data without a reference genome. Nat. Biotechnol..

[2] Matra D.D., Ritonga A.W., Natawijaya A., Poerwanto R., Sobir, Widodo W.D., Inoue E. (2019). Dataset of the first de novo transcriptome assembly of the arillode of Baccaurea motleyana. Data in Brief.

[3] Matra D.D., Ritonga A.W., Natawijaya A., Poerwanto R., Sobir, Widodo W.D., Inoue E. (2019). Dataset from de novo transcriptome assembly of Nephelium lappaceum aril. Data in Brief.

[4] Altschul S.F., Gish W., Miller W., Myers E.W., Lipman D.J. (1990). Basic local alignment search tool. J. Mol. Biol..

[5] Conesa A., Götz S., García-Gómez J.M., Terol J., Talón M., Blast2GO M. Robles (2005). A universal tool for annotation, visualization, and analysis in functional genomics research. Bioinformatics.

[6] Thiel T., Michalek W., Varshney R.K., Graner A. (2003). Exploiting EST databases for the development and characterization of gene-derived SSR-markers in barley (*Hordeum vulgare* L.), Theor. Appl. Genet..

